# Accelerating Digital Mental Health: The Society of Digital Psychiatry’s Three-Pronged Road Map for Education, Digital Navigators, and AI

**DOI:** 10.2196/84501

**Published:** 2025-11-27

**Authors:** John Torous, Kathryn Taylor Ledley, Carla Gorban, Gillian Strudwick, Julian Schwarz, Soumya Choudhary, Margaret Emerson, Michelle Patriquin, Allison Dempsey, Jason Bantjes, Laura Ospina-Pinillos, Jennie Hornick, Shruti Kochhar

**Affiliations:** 1 Division of Digital Psychiatry, Beth Israel Deaconess Medical Center, 330 Brookline Ave, Boston, MA, 02215, United States, 1 6176676700; 2 The Brain and Mind Centre, The University of Sydney, Camperdown, NSW, Australia; 3 Centre for Addiction and Mental Health, Toronto, ON, Canada; 4 Department of Psychiatry and Psychotherapy, Center for Mental Health, Immanuel Hospital Rüdersdorf, Brandenburg Medical School Theodor Fontane, Rüdersdorf, Germany; 5 Department of Psychiatry, National Institute of Mental Health and Neurosciences, Bangalore, India; 6 College of Nursing, University of Nebraska Medical Center, Ohama, NE, United States; 7 Department of Psychiatry and Behavioral Sciences at McGovern Medical School, UTHealth Houston, Houston, TX, United States; 8 Department of Psychiatry and Family Medicine, School of Medicine, Centers for American Indian and Alaska Native Health, Colorado School of Public Health, University of Colorado Anschutz Medical Campus, Aurora, CO, United States; 9 Department of Global Health, Faculty of Medicine and Health Sciences, Institute for Life Course Health Research, Stellenbosch University, Stellenbosch, South Africa; 10 Department of Psychiatry and Mental Health, Faculty of Medicine, Pontificia Universidad Javeriana, Bogota, Colombia; 11 JMIR Publications, Toronto, ON, Canada

**Keywords:** AI, LLM, mental health, apps, digital navigators, artificial intelligence, large language model

## Abstract

Digital mental health tools such as apps, virtual reality, and artificial intelligence (AI) hold great promise but continue to face barriers to widespread clinical adoption. The Society of Digital Psychiatry, in partnership with *JMIR Mental Health*, presents a 3-pronged road map to accelerate their safe, effective, and equitable implementation. First, education: integrate digital psychiatry into core training and professional development through a global webinar series, annual symposium, newsletter, and an updated open-access curriculum addressing AI and the evolving digital navigator role. Second, AI standards: develop transparent, actionable benchmarks and consensus guidance through initiatives like MindBench.ai to assess reasoning, safety, and representativeness across populations. Third, digital navigators: expand structured, train-the-trainer programs that enhance digital literacy, engagement, and workflow integration across diverse care settings, including low- and middle-income countries. Together, these pillars bridge research and practice, advancing digital psychiatry grounded in inclusivity, accountability, and measurable clinical impact.

## Background

As research on digital mental health tools, such as apps, virtual reality, and artificial intelligence (AI), continues to expand and suggest benefits, clinical interest has also increased, yet the gap between research and real-world implementation remains stark [1]. Toward bridging this divide, the Society of Digital Psychiatry (SODP) and *JMIR Mental Health* have developed a multipronged international approach to support training, implementation, and standards for digital mental health worldwide. Education and training must be complemented by workforce support and shared development of new digital and AI tools. Toward these aims, the SODP will support three pillars: (1) education and training through accessible learning activities, (2) workforce development through advancing support and standards for Digital Health Navigators, and (3) the development of clinical AI through creating actionable benchmarks and guidance.

The potential of digital mental health tools has become a global focus, with mental health now viewed as the single greatest health concern [2] and technology supporting this growing need [3]. With smartphone ownership now exceeding 90% in many countries and internet access improving across the world [4], technical barriers to digital mental health continue to fall. Although digital literacy barriers persist and are even greater for non-English speakers, they are now well-recognized, with programs demonstrating the capacity to support even patients with serious mental illness, adolescents navigating developmental transitions, and older adults.

Notably, however, even when reimbursement is available [5], multiple reviews have confirmed that adoption of digital solutions—ranging from mobile apps to digital therapeutics and AI—remains low among both patients and clinicians [6,7]. Patient self-help tools have also faced low engagement with data, suggesting that some degree of clinical support is necessary [8,9], especially since the smartphone app market is nonhomogeneous in providing privacy and efficacy information [10]. These challenges are not new, and landmark research by Bamul et al in 2019 [11] demonstrated that user engagement with mental health apps is negligible after 1 week. In 2019, Ng et al [12] also revealed that a lack of consensus in measuring engagement with and uptake of digital mental health tools precludes progress. The literature also points to inequities between high-income countries and low- and middle-income countries [13]. Despite the abundance of apps, only 14.5% are available in Spanish and none have undergone evaluation with Spanish-speaking users [14]. Moreover, digital competency—particularly patients’ and clinicians’ ability to effectively use and evaluate mental health apps—remains difficult to assess, as only a few validated instruments are currently available [15]. In 2015, Ben-Zeev et al [16] called for the need for a technology coach to support integration and engagement. Now, in 2025, these challenges remain largely unsolved, with engagement remaining low, standards nonexistent, and the use of technology coaches, now referred to as digital navigators [17], still minimal.

**Figure 1.**
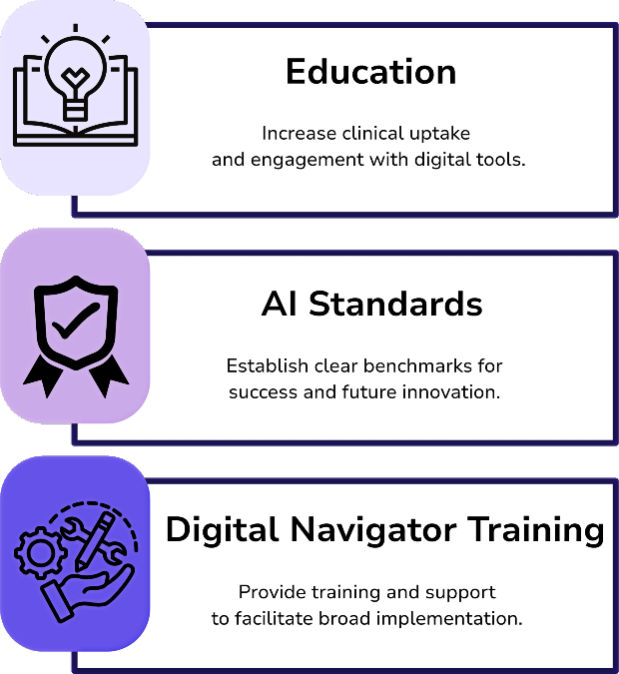
The Society of Digital Psychiatry’s 3-pronged approach. AI: artificial intelligence.

Although continued research will provide new solutions, additional approaches are necessary to ensure progress in digital mental health implementation can accelerate to meet the need for clinical care. The SODP is implementing a 3-pronged approach (Figure 1). First, expand education to increase clinical uptake and engagement with digital tools. Second, develop standards for AI to catalyze progress by establishing clear benchmarks for success and guiding future innovation. Lastly, provide training and support for digital navigators to facilitate broad implementation.

## Education

The SODP is dedicated to promoting clinician education in digital mental health. Today, many medical and nursing schools, peer specialist training programs, psychology and therapy degree programs, and other clinical training pathways do not cover digital mental health. Clinicians are understandably hesitant to recommend digital tools without adequate training or evidence-based guidance. Even when motivated to integrate technology, they frequently experience pressure to preserve existing workflows and not address ongoing barriers such as patient engagement and digital literacy, resulting in technology being deprioritized. These challenges are compounded by the lack of clinic- and system-level infrastructures to facilitate effective implementation [18].

*JMIR Mental Health* serves as the official journal of the SODP, embodying its shared mission to advance and disseminate cutting-edge research in digital mental health. So, in response to these challenges, the journal and society jointly host a monthly webinar series that highlights emerging innovations and diverse perspectives from invited speakers, featuring experts in digital psychiatry, internet interventions, cutting-edge technologies, mental health policy, ethics, and more [19]. Guests are thoughtfully selected based on their real-world work and ability to present up-to-date content. This series is designed to spotlight rigorous, critical, and forward-looking research that explores how and why new digital mental health tools and technologies work, their limitations, and their place within broader care models. This collaborative effort not only extends the reach of this research to a global audience but also enhances the society’s visibility and representation at major conferences, including the American Psychiatric Association annual meeting. These events provide a crucial platform for authors, editors, reviewers, and readers to engage with both *JMIR Mental Health* and SODP, learn about their initiatives, and contribute to the broader movement for evidence-based, peer-reviewed research. Complementary outreach activities, such as participation in the American Psychiatric Association’s Mental Health Innovation Zone and the publication of postevent blogs, further extend the journal and society’s reach, ensuring their work bridges the gap between research and clinical implementation. The recordings from these monthly webinars are subsequently repurposed into evergreen video content and made available asynchronously on YouTube and other social media platforms, including LinkedIn, X (formerly Twitter), Facebook, and Bluesky.

A similar approach is applied to the annual symposium, with each session produced and promoted individually to transform valuable knowledge into lasting, accessible educational resources. Furthermore, a quarterly newsletter copublished by *JMIR Mental Health* and the SODP provides insights into emerging trends and developments in digital mental health, featuring highlighted books, recent webinar recordings, upcoming industry events, and a quarterly question-and-answer section. This newsletter serves as a free, credible resource drafted by the SODP coleaders with citations and references for the 300 to 400 interprofessional members, ensuring they remain informed about advances in the field. As an immediate next step, the SODP will formally support the training and educational goals of clinicians and researchers by collaborating on a core curriculum with major educational bodies and providing a structured training plan for researchers, which is often required when applying for career development awards. The first step will be to release a new curriculum that updates the older one [20], with a focus on the expanded role of the Digital Health Navigator today as well as AI. Training will be accessible online, and coleaders will aim to offer local treatment in their region that will expand with time.

## AI Standards

The SODP is also committed to advancing the safe and effective application of AI to improve and expand mental health care. As AI and large language models become increasingly powerful and capable of enhancing aspects of clinical care, the need for clear benchmarks and standards is crucial [21]. Without effective ways to evaluate the risks and benefits of AI in mental health, the field will be unable to self-regulate and pursue the most promising advances. Today, there are over 50 frameworks to evaluate AI, yet none of them are actionable and able to guide care considerations or research advances [22-86]. Leveraging the global reach of the SODP, we will propose clear and actionable recommendations that will improve transparency, understanding, and collaboration in the mental health AI space. Importantly, these recommendations will emphasize the need for representativeness across populations—ensuring that datasets, evaluations, and implementations reflect the diversity of real-world users and contexts—to enhance fairness and generalizability. We will organize a Delphi consensus meeting with SODP members representing stakeholders from around the world. In addition, we will support the creation of benchmarking tools through the MindBench.ai initiative, which will enable any AI program to score against a representative, comprehensive, and rigorous battery of cases.

## Digital Navigator Training

Ultimately, the SODP is dedicated to promoting the growth of digital navigator roles. Digital navigators are currently conceptualized as human support coaches who can assist with digital access and equity, patient engagement, and the clinical integration of any digital health technology, are increasingly in demand [17,87-93]. However, foundational and scalable training, let alone certification and support for the role, is missing. The SODP will share leading examples of digital navigator programs worldwide to offer training and support through a theme issue in *JMIR Mental Health*, training on the SODP website, and training for the trainers by SODP coleaders. While each team will need to customize the role for their unique clinical setting and technology use case, there are core skills and competencies that the SODP will support to ensure consistent base training, fidelity to that training, and access to new training and skills as the field evolves while also exploring how best to adapt the digital navigator role into diverse health systems across the globe.

Education, standards, and digital navigator training are not panaceas that negate the need for more research. Instead, each of these 3 efforts can support new research advances by ensuring they reach clinicians quickly through education, are transparent about risks and benefits through standards, and are ready to be implemented through digital navigators. Thus, the ongoing partnership between the SODP and *JMIR Mental Health* provides an ideal platform to ensure that the best research informs the SODP’s efforts and its efforts to advance that research toward its goal of supporting care.

With this renewed focus, it is essential to note that the SODP’s name refers to the goal to support digital tools, including but not limited to therapy-based ones, and all patients, including those with severe mental illness. Such a goal requires an inclusive team, and the society’s educational focus already reflects this, as well as our leadership team, which represents different roles, professions, and voices from around the world. Looking ahead, the society is also committed to ensuring these efforts are relevant and actionable across the Global South by supporting culturally adapted AI standards, supporting scalable digital navigator training tailored for low- and middle-income country contexts, and fostering equitable research and policy partnerships worldwide.

Membership today stands between 300 and 400 interprofessional members and remains free at this time, with access at [94]. We hope you will join us in supporting the acceleration of digital mental health toward improving outcomes for all patients today and preventing these illnesses for people tomorrow.

## Abbreviations

AI: artificial intelligence
SODP: Society of Digital Psychiatry
